# Total polyphenolic (flavonoids) content and antioxidant capacity of different *Ziziphora clinopodioides* Lam. extracts

**DOI:** 10.4103/0973-1296.75904

**Published:** 2011

**Authors:** Shuge Tian, Yang Shi, Xiaoying Zhou, Liang Ge, Halmuart Upur

**Affiliations:** 1 *Xinjiang Key Laboratory of Famous Prescription and Science of Formulas, Xinjiang, People’s Republic of China*; 2 *College of TCM, Xinjiang Medical University, Xinjiang, People’s Republic of China*; 3 *College of Pharmacy, Xinjiang Medical University, Urumqi 830011, Xinjiang, People’s Republic of China*

**Keywords:** Antioxidant capacity, total flavonoids, total polyphenolic, *Ziziphora clinopodioides* Lam

## Abstract

**Objective:**

This paper investigates the total polyphenolic and flavonoid content as well as the antioxidant activity of *Ziziphora clinopodioides* Lam. extracts of different polarity.

**Materials and Methods:**

The total polyphenolic content was analysed using the Folin–Ciocalteu method. Total flavonoid content analysis was performed using the colorimetric method.

**Results:**

The total polyphenolic content of *Z. clinopodioides* is concentrated in parts of ethyl acetate (19.27%), chloroform (4.99%) and n-butanol extracts (3.94%) containing a small amount of the total polyphenolic content. The petroleum ether (0.23%) and ethanol extracts (1.64%) contain almost no polyphenolic content. The total flavonoid content of *Z. clinopodioides* is concentrated in parts of ethyl acetate (65.61%), chloroform (14.36%) and n-butanol extracts (10.76%) containing a small amount of the total polyphenolic content. The *Z. clinopodioides* Lam. ethyl acetate extract exhibits a good antioxidant activity.

**Conclusion:**

Ethyl acetate extracts contain a large number of polyphenolic compounds (19.27%) and flavonoids (65.61%) owing to good antioxidant capacity.

## INTRODUCTION

Free radicals produced by radiation, chemical reactions, and several redox reactions of various compounds may contribute to protein oxidation, DNA damage and lipid peroxidation in living tissues and cells.[1] Oxidative stress may be related to many disorders such as cancer, atherosclerosis, diabetes and liver cirrhosis.[2–4] Recent epidemiological studies have suggested that the increased consumption of whole grains, fruits and vegetables is associated with reduced risks of chronic diseases.[5] This association is attributed to the natural antioxidants such as vitamin C, tocopherol, carotenoids, polyphenolics and flavonoids which prevent free radical damage.[6] Free radical-induced oxidation of the human body is regarded as human ageing and the root cause of illnesses. Therefore, investigating the herbal anti-oxidation of the active ingredient has become a worldwide trend in botanical research.

*Ziziphora clinopodioides* is a traditional Uighur medicinal plant. This semi-perennial shrub-like herb grows in low hills, grassland, and arid slopes and is widely distributed in China, Mongolia, Turkey, Kazakhstan and Kyrgyzstan.[7] This plant is used mainly for the treatment of heart disease, high blood pressure, asthma hyperhidrosis, palpitation insomnia, oedema, cough, bronchitis, lung abscess and other diseases. Results of animal experiments show that *Z. clinopodioides* can significantly prolong the survival of the hypoxic mouse model and positively affect myocardial ischaemia and hypoxia.

Research on *Z. clinopodioides* focuses on the chemical constituents of volatile oil and antibacterial activity. Stability of the volatile oil has been studied in preliminary works but a survey of the different parts of polar solvent extraction of the total flavonoid and polyphenolic content and antioxidant activity is yet to be reported.

## MATERIALS AND METHODS

### Plant material

The plant (whole plant) used for the present study was collected from the Nan Mountains of China. Plant materials were further identified by Yonghe Li, a pharmacist from a Chinese medicine hospital of Xinjiang.

Rutin was obtained from National Institute for the Control of Pharmaceutical and Biological Products 100080 -200707; gallic acid monohydrate, ethanol, chloroform, *n*-butanol, ethyl acetate and petroleum ether were obtained from the Tianjin Reagent Co. (Tianjin, People’s Republic of China). All other solvents and chemicals were analytical grade and purchased from Tianjing Guangfu Chemical Ltd., Co, (Tianjin).

### Extraction process

The extract of dried and powdered plant (5 kg) was concentrated under decompression after impregnating with 90% ethanol extraction. Then using petroleum ether, chloroform, ethyl acetate, *n*-butanol and ethanol gradient to extract, concentrate the extracts, freeze-dried it into powder, spare.

### Polarity gradient extraction flowchart [Figure 1]

**Figure 1:**
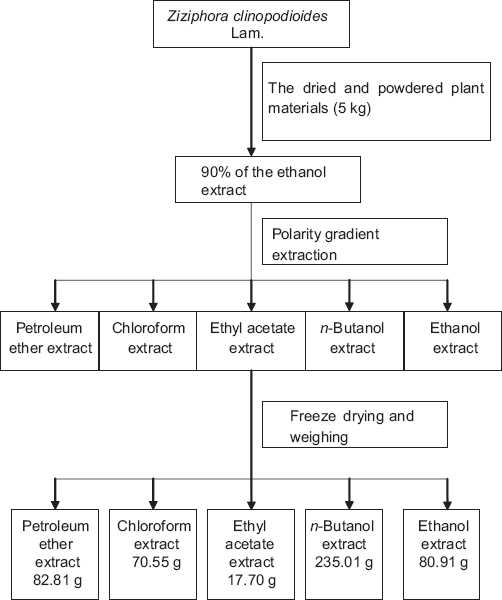
Polarity gradient extraction

### Determination of the total polyphenolic content

**Total polyphenolic content** (TPC) values from *Z. clinopodioides* petroleum ether, chloroform, ethyl acetate, *n*-butanol and ethanol extracts were determined according to the literature.[8] The various extracts were prepared as a 0.3 mg/ml aqueous solution. Each extract (0.5 ml) was mixed with 1 ml of Folin–Ciocalteu reagent (2 N) and was left to stand at room temperature for 1 min. The sodium carbonate (20% Na_2_ CO_3_) solution (2 ml) was added subsequently. Distilled water volume was increased to to 10 ml and the solution was allowed to stand at room temperature for 10 min. Supernatant absorbance was measured at 760 nm using a spectrophotometer (UV Cintra-40, GBC, Australia). The results were in contrast with the standards prepared similarly (with known gallic acid concentrations). All samples were analysed three times.

### Determination of the total flavonoid content

The total flavonoid content (TFC) was determined using a colorimetric method.[9] TPC values from *Z. clinopodioides* petroleum ether, chloroform, ethyl acetate, *n*-butanol and ethanol extracts were analysed using a colorimetric method. The various extracts were prepared as a 1 mg/ml 60% ethanol solution. Each extract (1 ml) was mixed with 0.4 ml of a 5% NaNO_2_ solution. The mixture was allowed to stay at room temperature for 6 min; 0.4 ml of a 10% AlCl_3_ × 9H_2_ O solution was added for 6 min followed by the addition of a 4 ml 4% NaOH solution. The ethanol solution (60%) was added to reach a final volume of 10 ml. The solution was mixed and kept at room temperature for 15 min. Absorbance was measured immediately against the prepared blank at 510 nm using a spectrophotometer (UV Cintra-40, GBC). Comparisons were made against standards prepared similarly (with known rutin concentrations). All samples were analysed three times.

### DPPH radical-scavenging capacity

The DPPH radical-scavenging (DPHH-RSC) activity of grains was estimated according to the method explained by Cheung, Cheung and Ooi[10] with some modifications. In this assay, antioxidants present in the sample reduced the DPPH radicals which have an absorption maximum at 517 nm. The DPPH radical solution was prepared by dissolving 12.4 mg DPPH in 1000 ml of ethanol (2 × 10^−5^ mol/l). First to occur is the extinction of the disposable cuvette with 1 ml of the ethanol DPPH solution. Ethanol (1.5 ml) was measured as blank. Then, *Z. clinopodioides* and the ethanol extract solutions were prepared at 1 mg/ml concentrations. Subsequently, add 1 mg/ml of extracts of different polarities (1 ml) along with 0.5 ml of anhydrous ethanol. The mixture was shaken vigorously and allowed to stand at room temperature in the dark for 30 min. The decrease in the absorbance of the resulting solution was monitored at 517 nm using a spectrophotometer (UV Cintra-40, GBC). The vitamin C standard solution (1 mg/ml) in ethanol was prepared under the same conditions.

### Superoxide anion-scavenging capacity

Superoxide anion production was measured by observing the autoxidation of epinephrine in terms of the rate of adrenochrome accumulation.[11] In this assay, 1 ml of the hydrochloric acid Tris-buffer solution (pH = 8.21, 0.05 mol/l) was held in a heating water bath at 25°C for 20 min. One millilitre (1 mg/ml) of the extracts of different polarities followed by 0.4 ml of pro-phloroglucinol (25 mmol/l) was added to the solution while holding in a heating water bath at 25°C for 5 min. Finally, 1 ml of the hydrochloric acid solution (8 mmol/l) was added and diluted 10 times as the sample. Absorbance at 320 nm was determined using a spectrophotometer (UV Cintra-40, GBC) using distilled water as the blank. A vitamin C standard solution (1 mg/ml) in distilled water was prepared under the same conditions.

### Hydroxyl radical-scavenging capacity

The assay was based on the benzoic acid hydroxylation method.[12] Hydroxyl radicals were generated by direct addition of iron (II) salts to a reaction mixture containing the phosphate buffer. In this assay sample, 0.2 ml of the pro-phenanthroline solution (7.5 mmol/l) was added to 2 ml of the phosphate buffer solution; 1 ml (1 mg/ml) of the extracts of different polarities, 1.4 ml of the hydrogen peroxide solution (1%) and 2.8 ml of distilled water were added to 0.2 ml of the ferrous sulphate solution and held in a heating water bath at 37°C for 1 h. Distilled water measured at 0.4 ml was also used as the damage liquid instead of the extracts of different polarities. Distilled water (0.8 ml) was used to serve as a blank. Absorbance at 510 nm was determined using a spectrophotometer (UV Cintra-40, GBC). A vitamin C standard solution (1 mg/ml) in distilled water was prepared under the same conditions.

## RESULTS AND DISCUSSION

Table 1 shows that the total polyphenolic content of *Z. clinopodioides* is concentrated in parts of ethyl acetate (19.27%), chloroform (4.99%) and *n*-butanol extracts (3.94%) containing a small amount of the total polyphenolic content. The petroleum ether (0.23%) and ethanol extracts (1.64%) contain almost no polyphenolic content. This is due to the total polyphenolic compounds resulting from polarity, low polarity of the total polyphenolic concentration in chloroform extracts and high polarity of the total polyphenolic concentration in *n*-butanol extracts. The total flavonoid content of *Z. clinopodioides* is concentrated in parts of ethyl acetate (65.61%), chloroform (14.36%) and *n*-butanol extracts (10.76%) containing a small amount of the total polyphenolic content. Large absorbance is due to the petroleum ether extract-containing pigment making the solution turbid.

**Table 1:** Total polyphenolic content and total flavonoid content

Different extraction layers	TPC (%, X ± S, n = 3)	TFC (%, X ± S, n = 3)
Petroleum ether	0.230 ± 0.003	11.750 ± 0.155
Chloroform	4.990 ± 0.062	14.360 ± 0.178
Ethyl acetate	19.270 ± 0.266	65.610 ± 0.826
*n*-Butanol	3.940 ± 0.068	10.760 ± 0.132
Ethanol	1.640 ± 0.031	3.770 ± 0.058

Table 2 shows the clear difference in antioxidant activities. The ethyl acetate extract contains a large number of polyphenolic compounds and flavonoids mainly due to the polarity of polyphenols determined. Polyphenolic compounds are one of the most effective antioxidative constituents in fruits, vegetables and grains.[13] Hence, it is important to quantify polyphenolic contents and to assess the contribution to the antioxidant activity.

**Table 2:** DPPH radical-scavenging capacity (DPPH-RSC), superoxide anion-scavenging capacity (superoxide-ASC) and hydroxyl radical-scavenging capacity (hydroxyl -RSC) in different polar solvents

Different extraction layer	DPPH-RSC (%, X ± S, n = 3)	Superoxide-ASC (%, X ± S, n = 3)	Hydroxyl-RSC (%, X ± S, n = 3)
Petroleum ether	6.97 ± 0.09	4.75 ± 0.07	7.40 0.14
Chloroform	25.69 ± 0.35	20.16 ± 0.32	14.33 ± 0.36
Ethyl acetate	34.11 ± 0.54	40.12 ± 0.71	98.27 1.43
n-Butanol	28.27 ± 0.41	35.82 0.53	26.14 ± 0.42
Ethanol	21.48 ± 0.31	29.94 ± 0.49	15.91 0.24
Vitamin C	32.53 ± 0.42	33.54 ± 0.54	50.01 ± 0.79

DPPH-RAS (%) = (*A*_blank_ − *A*_sample_)/*A*_blank_×100%, Superoxide-RAS (%) = (*A*_blank_ − *A*_sample_)/*A*_blank_×100%, Hydroxyl-RAS (%) = (*A*_sample_ − *A*_damage_)/(*A*_blank_ − *A*_damage_)×100%

No literature has been found regarding the total polyphenolic content and antioxidant capacity of different *Z. clinopodioides Lam*. extracts. The results obtained herein are in agreement to a certain degree with the traditional uses of *Z. clinopodioides Lam*. as a valuable source for antioxidant drugs.

The *Z. clinopodioides Lam*. ethyl acetate extract exhibits a good antioxidant activity. This also proves that polyphenolic compounds and antioxidant activities are closely linked. This is the first study to provide determination data on different polarity extracts of *Z. clinopodioides Lam*.
